# Understanding Viral Haemorrhagic Fevers: Virus Diversity, Vector Ecology, and Public Health Strategies

**DOI:** 10.3390/pathogens13100909

**Published:** 2024-10-18

**Authors:** Roger Hewson

**Affiliations:** 1 Department of Infection Biology, London School of Hygiene & Tropical Medicine, Keppel Street, London WC1E 7HT, UK; roger.hewson1@lshtm.ac.uk; 2 Virus Reference & Research (Special Pathogens), WHO—Collaborating Centre, Salisbury SP4 0JG, UK; 3 UK—Health Security Agency, Porton Down, Salisbury SP4 0JG, UK

**Keywords:** *Arenaviridae*, *Filoviridae*, *Flaviviridae*, *Hantaviridae*, *Nairoviridae*, transmission vectors, zoonotic, surveillance, ecology, interdisciplinary approaches, global cooperation

## Abstract

Viral haemorrhagic fevers encompass a diverse group of severe, often life-threatening illnesses caused by viruses from multiple families, including *Arenaviridae, Filoviridae, Flaviviridae*, *Hantaviridae*, *Nairoviridae*, *Peribunyaviridae*, and *Phenuiviridae*. Characterised by fever and haemorrhagic symptoms, these diseases challenge public health systems by overwhelming healthcare facilities, complicating diagnostic processes, and requiring extensive resources for containment and treatment, especially in resource-limited settings. This discussion explores the intricate relationships between VHFs and their transmission vectors—both animal and arthropod—and examines the impact of ecological and geographic factors on disease spread. The primary transmission of VHFs typically occurs through direct contact with infected animals or via bites from haematophagous arthropods, facilitating zoonotic and, at times, human-to-human transmission. With an emphasis on the role of diverse wildlife, domesticated animals, and vectors such as mosquitoes and ticks in the epidemiology of VHFs, there is a recognised need for robust surveillance and strategic public health responses to manage outbreaks. This review discusses the necessity of interdisciplinary approaches that integrate virology, ecology, and public health to enhance diagnostic capabilities, develop vaccines and antivirals, and improve outbreak interventions. Exploring the ecological and biological dynamics of VHFs will help bolster a deeper understanding of these emerging viruses and underpin preparation for future outbreaks. The importance of enhanced global cooperation, continuous research, and collaboration to mitigate the public health threats posed by these complex infections is a central theme, serving as a foundational strategy to reinforce worldwide preparedness and response efforts. Future directions include addressing gaps in vaccine development and tailoring public health strategies to the unique challenges of managing VHFs, such as the rapid mutation rates of viruses, the need for cold chain logistics for vaccine distribution, and socio-economic barriers to healthcare access, in order to ensure readiness for and effective response to emerging threats worldwide.

## 1. Introduction to Viral Haemorrhagic Fevers and Their Diverse Aetiology

Viral haemorrhagic fevers (VHFs) encompass a group of severe and often life-threatening illnesses caused by a diverse array of viruses from families including *Arenaviridae*, *Filoviridae*, *Flaviviridae*, *Hantaviridae*, *Nairoviridae*, *Peribunyaviridae*, and *Phenuiviridae*. This manuscript focuses on VHFs that have significant public health impacts due to their high mortality rates and potential for causing outbreaks. VHFs are a pressing global health concern due to their potential for rapid outbreaks, high mortality rates, and complex transmission dynamics involving zoonotic hosts and arthropod vectors. Despite advances in our understanding of individual VHF-causing viruses, significant gaps remain in integrating knowledge of viral diversity, ecology, and the public health strategies needed to effectively control these diseases. This review aims to provide a comprehensive analysis of VHFs by exploring the interactions between virus evolution, ecological changes, and the role of animal vectors and reservoirs in disease transmission. Furthermore, it emphasises the importance of interdisciplinary approaches, including One Health initiatives, to enhance diagnostic capabilities, improve surveillance, and inform vaccine and treatment development. By addressing the intricate ecology of VHFs, this review seeks to motivate future research and foster global collaboration to better prepare for emerging threats.

Characterised by fever and bleeding disorders, these viruses inflict significant damage on multiple organ systems, particularly the cardiovascular system, impairing the body’s overall function [1]. VHFs are notorious for their rapid progression and high mortality rates in the absence of supportive care. While some VHFs cause relatively mild illness, others lead to severe life-threatening conditions. The substantial genetic diversity among these viruses complicates their transmission dynamics and clinical manifestations, and poses challenges in the development of diagnostics and vaccines.

Viral diversity in haemorrhagic fevers encompasses the genetic, structural, and ecological variations that affect virus virulence, immune system evasion, and host interactions, further influencing the epidemiological patterns of outbreaks across different environments and host species. This diversity is typically confined to specific geographical regions influenced by climate, vector habitats, and human interaction [2,3], with VHFs primarily transmitted through direct contact with infected animals or via hematophagous arthropods. These arthropod vectors are crucial in spreading infections to new hosts through their blood-feeding activities, facilitating not only zoonotic transmission but also the potential for sustained human-to-human spread. The central aim of this review is to explore the complex interplay between the diverse viral agents of VHFs, considering the role of climate change in altering vector habitats and the impact of deforestation on wildlife reservoirs, and their ecological and geographical contexts to underscore the challenges in disease prediction, control, and management. It advocates for an integrated approach that combines virology, ecology, and public health strategies to enhance surveillance, develop targeted interventions, and prepare for future outbreaks. This review will provide a detailed examination of viral diversity and its implications in order to drive advancements in global health responses and research methodologies. A list of viruses known to cause VHFs is included in Table 1, illustrating the scope of this challenge and the necessity for ongoing research and international cooperation.

### 1.1. Strategic Public Health Responses to Viral Haemorrhagic Fever Diversity

The vast diversity of VHF viruses—spanning different viral families, transmission routes, and wildlife reservoirs, with some involving arthropod vectors—plays a pivotal role in shaping public health strategies. These strategies include prevention, control, treatment, and clinical management, while also driving scientific research [4]. These viruses, with their various transmission dynamics, reservoir hosts, and geographical distributions, require robust surveillance systems to detect and manage outbreaks effectively [5]. The clinical symptoms of different VHF viruses can vary widely, from mild to life-threatening, highlighting the need for in-depth knowledge to support timely public health responses, including accurate diagnosis, appropriate medical interventions, and the development of targeted treatments.

Transmission through arthropod vectors such as mosquitoes or ticks, as well as zoonotic origins, emphasises the need to understand the specific vectors, hosts, and environmental factors involved in transmission. Such consideration is important for designing and implementing targeted vector control strategies. Additionally, recognising the diversity among VHFs helps assess the risks of cross-species transmission and supports the development of strategies to prevent spill-over events [6]. A ‘One Health’ approach is recommended to tackle these challenges, as it integrates insights from virology, ecology, and public health, enhancing our understanding of the complex interactions among viruses, animal hosts, vectors, and environmental factors [7].

By fostering an interdisciplinary strategy, we can improve prevention, detection, and control measures against these diseases. For instance, comprehensive knowledge of VHF diversity enables health protection centres and international organisations to better anticipate and mitigate potential threats, strengthening global health security. This strategic knowledge is also essential in guiding scientific research towards the development of new diagnostics, antiviral drugs, and vaccines, driving innovation and improving our response to emerging infectious diseases [5].

### 1.2. The Role of Animal and Arthropod Vectors in VHF Transmission

Animals play an integral role in the transmission dynamics of VHFs, serving as primary reservoirs where viruses can replicate and persist without causing noticeable illness—an essential factor for the virus’s survival in the ecosystem [8]. Direct transmission from animals to humans, accounting for approximately 70% of initial infection cases, is a major pathway for the spread of disease. This often occurs through human contact with the bodily fluids of infected animals, such as blood, saliva, or urine [9]. Such exposures are common during activities like handling, slaughtering, or consuming infected animals, especially in regions where humans frequently interact with wildlife or domestic animals [10].

Additionally, VHFs are often transmitted to humans via arthropod vectors like ticks and mosquitoes. These vectors carry the virus after a period of replication and maturation, transmitting it to humans through bites. The involvement of arthropods adds complexity into the control and prevention of VHFs, necessitating a deep understanding of the life cycles and behaviours of both animal reservoirs and their arthropod vectors [11]. The following sections will explore the roles of specific animal groups—such as rodents, bats, non-human primates, and arthropods—as vectors and reservoirs for the key viruses under consideration. An overview of these roles is provided in Table 1.

**Table 1:** Overview of the major VHF viruses, categorising them by their respective virus families. Information is given about the natural reservoirs or hosts and vector relationships where relevant for each virus, the primary routes through which each virus is transmitted to humans, their geographical distribution, and the availability of vaccines against them. * Clinical features of hantavirus infections are generally separated by distinct virus tropism in either the heart and lungs for HCPS or the kidneys for HFRS. However, petechia, haemorrhage, and renal disfunction can be observed in HPS, and pulmonary involvement can be seen in HFRS cases. Hence, the broad term hantavirus disease has been proposed to better describe the disease condition [12]. ** Dengue fever is an acute illness caused by four serotypes of dengue virus (DENV-1 to DENV-4). Infections can range from asymptomatic or mild fever to severe dengue, which includes plasma leakage, haemorrhage and organ damage [13]. A second infection with a different serotype can lead to more severe disease due to a process called antibody-dependent enhancement, where antibodies from the first infection worsen the immune response, increasing the risk of haemorrhagic symptoms [14]. *** *Henipaviruses*, Nipah, and Hendra are not traditionally classified as VHFs, but are included in the table due to their potential for causing severe zoonotic diseases.

Virus Family	Virus Name	Primary Reservoirs	Arthropod Vector	Predominant Transmission Route	Geographic Distribution	Disease Features	Vaccine Availability
*Filoviridae*	*Zaire ebolavirus*	Fruit bats, primates	-	Direct contact with body fluids	Sub-Saharan Africa	Severe haemorrhagic fever, high fatality rate	Licensed vaccines available
*Filoviridae*	*Sudan ebolavirus*	Fruit bats, primates	-	Direct contact with body fluids	Sub-Saharan Africa	Severe haemorrhagic fever, high fatality rate	No specific licensed vaccine available
*Filoviridae*	*Taï Forest* *ebolavirus*	Fruit bats, primates	-	Direct contact with body fluids	Sub-Saharan Africa (Côte d’lvoire)	Fever, but not well understood (1 non-fatal case)	No specific licensed vaccine available
*Filoviridae*	*Bundibugyo ebolavirus*	Fruit bats, primates	-	Direct contact with body fluids	Sub-Saharan Africa (Uganda)	Moderate haemorrhagic fever	No specific licensed vaccine available
*Filoviridae*	*Reston ebolavirus*	Non-human primates, pigs	-	Direct contact with body fluids	Philippines	Non-pathogenic in humans	No specific licensed vaccine available
*Filoviridae*	*Bombali ebolavirus*	Fruit bats, primates	-	Direct contact with body fluids	Sub-Saharan Africa (Sierra Leone)	No evidence of human disease	No specific licensed vaccine available
*Filoviridae*	*Marburg marburgvirus*	Primates, fruit bats/*Rousettus aegyptiacus*	-	Direct contact with body fluids	Sub-Saharan Africa	Similar to Ebola, very high fatality rate	No licensed vaccine
*Arenaviridae*	*Lassa mammarenavirus*	*Mastomys natalensis*	-	Contact with infected rodent excreta	West Africa	Haemorrhagic fever with renal syndrome, deafness in survivors	Vaccines in development
*Arenaviridae*	*Lujo mammarenavirus*	Unknown		Contact with infected rodent excreta	Zambia, South Africa	Haemorrhagic fever	No vaccine
*Arenaviridae*	*Junin mammarenavirus*	Rodents/Calomys *musculinus,* and *C laucha*	-	Aerosol particles, direct contact	Argentina	Argentine haemorrhagic fever, neurological and haemorrhagic symptoms	Licensed vaccine in Argentina
*Arenaviridae*	*Guanarito mammarenavirus*	Rodents/*Zygodontomys brevicauda*	-	Contact with infected rodent excreta	Venezuela	Venezuelan haemorrhagic fever, severe symptoms	No licensed vaccine
*Arenaviridae*	*Chapare mammarenavirus*	Cricetidae rodents, possibly *Oligoryzomys microtis*	-	Contact with bodily fluids	Bolivia	Chapare haemorrhagic fever, similar to Ebola	No licensed vaccine
*Arenaviridae*	*Machupo mammarenavirus*	Rodents/*Calomys callosus*	-	Contact with infected rodent excreta	Bolivia	Bolivian haemorrhagic fever, severe haemorrhagic signs	No licensed vaccine
*Arenaviridae*	*Sabia mammarenavirus*	As yet unidentified *Cricetidae* rodents	-	Contact with infected rodent excreta	Brazil	Brazilian haemorrhagic fever, severe symptoms	No licensed vaccine
*Peribunyaviridae*	*Ngari othobunyavirus*	Livestock (cattle, sheep, and goats)	*Aedes, Anopheles* and *Culex* species	Mosquito bites	Sub-Saharan Africa (Kenya)	Moderate haemorrhagic fever	No licensed vaccine
*Hantaviridae*	*Andes orthohantavirus*	*Oligoryzomys longicaudatus*	-	Contact with infected rodent excreta	South America	HCPS *	No licensed vaccine
*Hantaviridae*	*Bayou orthohantavirus*	*Oryzomys palustris*	-	Contact with infected rodent excreta	Southern USA	HCPS *	No licensed vaccine
*Hantaviridae*	*Black Creek Canal orthohantavirus*	*Sigmodon hispidus*	-	Contact with infected rodent excreta	Southern USA (Florida)	HCPS *	No licensed vaccine
*Hantaviridae*	*Chocló orthohantavirus*	*Oligoryzomys longicaudatus*	-	Contact with infected rodent excreta	Central America (Columbia)	HCPS *	No licensed vaccine
*Hantaviridae*	*Dobrava orthohantaviru* *s*	*Apodemus flavicollis*	-	Contact with infected rodent excreta	Central Europe	Haemorrhagic fever with renal syndrome (HFRS) *	No licensed vaccine
*Hantaviridae*	*Hantaan orthohan* *taviru* *s*	*Apodemus agrarius*	-	Contact with infected rodent excreta	Asia	HFRS *	Hantavax licenced vaccine in South Korea
*Hantaviridae*	*LagunaNegra orthohantavirus*	*Calomys callosus*	-	Contact with infected rodent excreta	South America (Paraguay, Bolivia, Argentina)	HCPS *	No licensed vaccine
*Hantaviridae*	*Sin Nombre orthohantavirus*	*Peromyscus maniculatus*	-	Contact with infected rodent excreta	North America	HCPS *	No licensed vaccine
*Hantaviridae*	*Seoul orthohantavirus*	*Rattus norvegicus*	-	Contact with infected rodent excreta	World-wide	HFRS *	No licensed vaccine
*Nairoviridae*	*Crimean–Congo haemorrhagic fever (CCHF) orthonairovirus*	Ticks, ruminants, and livestock	*Hyalomma* ticks	Tick bites, contact with infected animals	Africa, Asia, Eastern Europe	Acute fever, haemorrhage, high fatality rate	No licensed vaccine
*Flaviviridae*	Yellow fever virus	Primates, mosquitoes	*Aedes aegypti, Ae albopictus, Haemagogus janthionomys, Haemogogus leucocelaenus*	Mosquito bites	Africa, South America	Acute viral haemorrhagic disease, jaundice	Effective vaccine available
*Flaviviridae*	Dengue viruses **	Primates, mosquitoes	*Aedes aegypti*	Mosquito bites	Worldwide	Asymptomatic to mild fever. Occasionally haemorrhagic fever **	Licenced vaccine available
*Flaviviridae*	Kyasanur forest disease virus	Small mammals	*Haemaphysalis spinagera*	Tick bites	India, especially Karnataka	Fever, haemorrhage encephalitis gastrointis	No licensed vaccine
*Flaviviridae*	Omsk haemorrhagic fever virus	Rodents, ticks	*Dermacentor reticulatus*	Tick bites	Siberia, Russia	Fever, haemorrhagic symptoms, neck stiffness	No licensed vaccine
*Flaviviridae*	Alkhurma hemorrhagic fever virus	Ticks, livestock	*Ornithodoros savignyi*	Tick bites	Middle East, especially Saudi Arabia	Fever, haemorrhagic symptoms, encephalitis	No licensed vaccine
*Phenuiviridae*	Rift Valley fever virus	Livestock (cattle, sheep, and goats)	*Aedes* and *Culex* genera	Mosquito bites, contact with infected blood	Sub-Saharan Africa, Middle East	Acute fever, liver abnormalities haemorrhagic fever	Vaccine available for livestock, none for humans
*Phenuiviridae*	Severe fever with thrombocytopenia syndrome virus	Ticks, deer livestock	*Haemaphysalis longicornis*	Tick bites	China Japan S Korea	Haemorrhagic, leukopenia	No licensed vaccine
*Paramyxoviridae*	Nipah virus ***	Pteropus lylei *P. vampyrus* and *P. hypomelanus* *P. medius*	-	Direct contact with infected urine, respiratory secretions	SE Asia	Fever, respiratory symptoms, and encephalitis (not a VHF) ***	No licensed vaccine
*Paramyxoviridae*	Hendra virus ***	*Pteropus alecto* and *P. conspicillatus*	-	Direct contact with infected urine, respiratory secretions	Australasia	Fever, respiratory symptoms, and encephalitis (not a VHF) ***	No licensed vaccine

### 1.3. Rodent Reservoirs

Rodents, particularly those from the *Muridae* and *Cricetidae* families, play an important role as reservoir hosts for a variety of Old World and New World viruses, respectively. These rodent families have adapted to a wide range of environments across the globe, reflecting their significant impact on public health due to their wide distribution and close proximity to human populations. Multiple species of rats and mice within these families are natural reservoirs for a variety of arenaviruses that are aetiological agents for VHFs, such as *Lassa mammarenvirus* (LASV), responsible for Lassa fever endemic in West Africa [15]; *Junín mammarenavirus* (JUNV), responsible for Argentine haemorrhagic fever [16]; *Guanarito mammarenavirus* (GTOV), responsible for Venezuelan haemorrhagic fever [17]; *Machupo mammarenavirus* (MACV) and *Chapare mammarenavirus* (CHAPV), which trigger respective [18,19] haemorrhagic fevers in Bolivia; *Sabia mammarenavirus* (SABV), responsible for Brazilian haemorrhagic fever [20]; and *Lujo mammarenavirus* (LUJV), responsible for Lujo haemorrhagic fever [21] in Zambia and South Africa. Each virus is associated with a distinct rodent host and geographic distribution. For example, LASV is primarily carried by the multimammate mouse (*Mastomys natalensis*) in West Africa, while Junín virus is hosted by the corn mouse (*Calomys musculinus*), prevalent in agricultural regions of Argentina where Argentine haemorrhagic fever is endemic.

Additionally, the *Muridae* family also harbours various strains of hantaviruses, which are responsible for haemorrhagic fever with renal syndrome (HFRS) in Eurasia and Hantavirus Cardio-Pulmonary Syndrome (HCPS) in the Americas. Notable examples include *Hantaan orthohantavirus* (HTNV) [22], *Dobrava orthohantavirus* (DOBV) [23], and *Seoul orthohantavirus* (SEOV) [24], all primarily associated with HFRS, showcasing a haemorrhagic pattern of kidney damage in affected individuals. Conversely, *Sin Nombre orthohantavirus* (SNV) [25] in North America and Andes *orthohantavirus* (ANDV) [26] in South America are notorious for causing HCPS, a severe respiratory disease that can occasionally involve haemorrhagic symptoms [27]. This variation in symptoms has led to the broader term “hantavirus disease”, reflecting the wide range of clinical outcomes associated with hantavirus infections.

Transmission to humans typically occurs through direct contact with rodent excreta [28], handling infected rodents [29], or, in rare cases, nosocomial events [30]. Environmental changes such as urbanisation [31] and agricultural practices [32] can increase human exposure to these rodents, raising the risk of zoonotic transmission. This highlights the need for public health interventions focused on reducing human–rodent interactions, such as improving waste management to limit rodent habitats, implementing effective rodent control strategies, and educating communities on the risks of rodent exposure.

Comprehensive surveillance systems that monitor the systematic collection, analysis, and interpretation of data on both rodent populations and human health cases are important for early detection and response to outbreaks, helping to prevent widespread transmission. The complex interplay between rodent behaviour, their ecological niches, and human activities underscores the need for an integrated approach to managing rodent-borne VHFs that includes ecological studies, public health interventions, and community engagement.

### 1.4. Bat Reservoirs

Bats play a critical role as natural reservoirs for various VHFs, particularly those caused by members of the *Filoviridae* family, which includes viruses such as Ebola and Marburg. Their unique ability to host these viruses without showing signs of disease allows these viruses to persist within bat populations over long periods, contributing to the complexity of filovirus disease ecology and transmission dynamics.

The Egyptian fruit bat, *Rousettus aegyptiacus*, commonly found in Africa, has been extensively studied and shown to be a primary reservoir for the Marburg virus (MARV) [33]. Studies consistently finding Marburg virus RNA in tissues of these bats, aligned with the geographic occurrence of outbreaks, underscore their significant role in the maintenance and transmission of the virus to humans—often through direct contact or indirectly via contaminated sites.

In contrast, establishing a definitive link between specific bat species and Ebola viruses has been more challenging due to the elusive nature of the virus within bat hosts. While multiple species of fruit bats have been implicated as potential reservoirs [34] through the detection of viral nucleic acids and antibodies suggesting their role in the viral epidemiological cycle, definitive evidence of the involvement of bats in the maintenance of Ebola viruses is still absent. Therefore, this suggests that other hosts may play roles in maintaining the virus within forest ecosystems [35]. This alternative hypothesis underscores the need for broader ecological studies to understand potential maintenance hosts or community interactions that could influence viral persistence and spillover.

Expanding our understanding of how filoviruses are maintained in bat populations is key to preventing and controlling outbreaks of VHFs and other emerging viruses [36]. Ongoing research is focused on the ecological and biological factors that facilitate virus persistence in bats, the interaction between bats and human environments, and the conditions that lead to virus spillover. This research is crucial not only for disease prevention, but also for informing public health strategies to mitigate the risk of future zoonotic outbreaks. Enhanced surveillance of bat populations, particularly in regions prone to Ebola and Marburg disease outbreaks, and community education on avoiding contact with bats are essential components of these strategies.

### 1.5. Other Vertebrate Reservoirs and Hosts

Non-human primates, such as monkeys and apes, play a significant role in the ecology of certain VHFs, acting as both reservoirs and amplifiers of these diseases. For example, monkeys infected with the yellow fever virus (YFV) can perform dual roles by sustaining the virus within their populations and facilitating its spread to humans through mosquito bites. This interspecies virus transmission cycle is key to the maintenance and spread of yellow fever, particularly in forested areas of Africa and South America where sylvatic cycles include specific species of mosquitoes and non-human primates.

The transmission of VHFs such as Ebola and Marburg also occurs through direct human contact with infected non-human primates. This is common in regions where cultural practices include the hunting, butchering, and consumption of bushmeat, posing significant risks for virus spillover to humans and potentially sparking outbreaks.

The interface between non-human primates and humans is further complicated by environmental challenges such as deforestation and habitat encroachment, as well as socio-economic challenges such as poverty and reliance on bushmeat for food. Activities like deforestation, habitat encroachment, and urbanisation bring humans into closer contact with primate habitats, increasing the risk of disease transmission. The economic imperatives of the bushmeat trade often exacerbate this contact, making it difficult to mitigate transmission risks.

Addressing these challenges requires a comprehensive strategy that includes the rigorous surveillance and monitoring of primate populations, public health education on the dangers of bushmeat consumption, and the implementation of safe handling practices. Conservation efforts aimed at protecting primate habitats and maintaining natural barriers to disease transmission are equally crucial. Strengthening international cooperation and fostering community engagement are essential for effectively managing the health risks associated with VHFs in regions where these interactions are most pronounced.

### 1.6. Role of Arthropod Vectors

Arthropods, notably mosquitoes and ticks, play a significant role in the transmission of various VHFs. Mosquitoes, notably from the *Aedes* and *Culex* genera [37,38], are primary vectors for RVFV, transmitting this virus to humans through blood-feeding activities, while YFV is predominantly spread by *Aedes* mosquitoes. *Aedes* mosquitoes, known for their preference for human blood and adaptability to various environments, thrive in both urban and rural settings. Their efficiency in transmitting viruses is heightened by their ability to breed in stagnant water [39], which is often found in human-made habitats such as discarded tires and artificial water storage containers. These breeding sites, particularly common in densely populated areas, significantly increase the risk of disease transmission, making *Aedes* mosquitoes a key vector in the spread of viral diseases.

Conversely, *Culex* mosquitoes, which are also found in urban and rural areas, transmit viruses over long distances by developing in a wide range of habitats, including larger bodies of stagnant water. They feed on a variety of hosts, including birds and humans, facilitating transmission cycles that involve multiple species, spanning ecosystems and large geographical regions [40]. This broad host range and habitat flexibility make *Culex* mosquitoes effective vectors for several arboviruses, including transmission cycles that can bridge wildlife and human populations.

Both genera contribute to the spread of VHFs by facilitating zoonotic and anthroponotic transmissions, underscoring the need for targeted vector control strategies that consider the specific breeding habitats and feeding preferences of these mosquito species. Enhanced surveillance and adaptive public health strategies are essential to mitigate the impact of these vectors on the spread of VHFs.

Ticks, on the other hand, live longer than mosquitoes, and viruses can persist and replicate within the tick’s tissues throughout their lifetime. This enables transmission across multiple blood meals, permitting ticks to serve as both reservoirs and vectors [41] for several VHF viruses. For instance, CCHFV is primarily transmitted by *Hyalomma* genus ticks [42]. Other examples include Kyasanur forest disease virus (KFDV) transmitted by *Haemaphysalis spinigera* [43], severe fever with thrombocytopenia syndrome virus (SFTSV) transmitted by *Haemaphysalis longicornis* [44], Alkhurma haemorrhagic fever virus (AHFV) transmitted by *Ornithodoros savignyi* [45], and Omsk haemorrhagic fever virus (OHFV) transmitted by *Dermacentor reticulatus* [46], illustrating the specificity of vector–virus relationships.

The specificity of vector–host interactions in tick-borne diseases is shaped by ecological factors such as temperature, humidity, and vegetation cover, all of which influence the epidemiological risks associated with ticks. Understanding tick life cycles, habitat preferences, and feeding behaviours is essential for reducing the risk of tick-borne diseases [47]. Climate change adds further complexity to these dynamics, likely altering the distribution and activity periods of ticks, expanding their geographical range and increasing the transmission window of tick-borne diseases [48]. These factors collectively contribute to the intricate ecology of tick-borne viruses, necessitating adaptive management strategies to address the increased risk to human health.

Similarly, mosquito-borne viruses exhibit transmission dynamics shaped by ecological factors unique to mosquitoes. The life cycles of mosquitoes, particularly their aquatic stages, are influenced by the availability of water bodies, with different species showing preferences for various water qualities. Climate change also plays a significant role in affecting mosquito behaviour, distribution, and population dynamics. Temperature increases enhance mosquito development rates, increase biting rates, and shorten the pathogen incubation period within mosquitoes, with ecological modelling studies illustrating how such factors may boost their capacity to transmit viruses such as YFV [49]. Additionally, changes in precipitation patterns impact mosquito breeding site availability, directly affecting population sizes and disease outbreak potential.

Urbanisation and changes in land use also create new breeding sites for mosquitoes, increase exposure, and facilitate the spread of mosquito-borne diseases. Therefore, public health strategies must evolve to include integrated mosquito management practices, encompassing environmental management, biological control, and the use of insecticides, to effectively combat the spread of these diseases.

Robust surveillance networks that oversee the organised gathering, evaluation, and interpretation of information on both arthropod vector populations and human health incidents are essential for early detection and response to outbreaks, helping to prevent widespread transmission. The elaborate relationships between vector habits, their environmental niches, and human actions highlights the necessity for an inclusive strategy in managing vector-borne VHFs, incorporating ecological research, public health measures, and community involvement. Effective surveillance systems are essential for preparing public health systems to respond to arbovirus outbreaks, ensuring timely interventions and mitigating the impact of these diseases [50,51,52].

## 2. Geographical Distribution of VHF Viruses

### 2.1. Factors Influencing the Spread of VHF Viruses

The geographical distribution of VHF viruses is principally influenced by the ecological, climatic, and human factors that affect the presence and spread of their vectors and reservoirs. These factors play an important role in the frequency and scale of VHF outbreaks.

### 2.2. Regional Overviews

#### 2.2.1. Africa

The frequent contact between humans and infected animal hosts or arthropod vectors in Africa significantly increases the risks and spread of VHFs, marking the continent as a significant hotspot for these diseases. VHFs like yellow fever (YF), Rift Valley fever (RVF), Crimean–Congo haemorrhagic fever (CCHF), Lassa fever (LF), Marburg virus disease (MVD), and Ebola virus disease (EVD) have all had outbreaks in Africa. These diseases manifest with high mortality rates and are characterised by rapid onset and severe symptoms, including fever and haemorrhagic manifestations [53].

In regions where these diseases are endemic, outbreaks are often triggered by interactions with infected wildlife or through vectors such as mosquitoes and ticks. For example, YF, endemic in the tropical areas of sub-Saharan Africa (and also in parts of South America) is transmitted by *Aedes* mosquitoes, which thrive in both urban and jungle cycles, affecting both humans and monkeys. RVF, primarily affecting livestock and occasionally humans, spreads through *Aedes* mosquitoes and is exacerbated by climatic conditions like heavy rainfall and flooding, which increase the opportunities for mosquito breeding.

The relationship between human activities and the natural habitats of these vectors often leads to increased transmission risks. For example, deforestation and expanding agricultural frontiers increase human exposure to tick habitats, escalating the spread of diseases like CCHF. Similarly, LF transmits through contact with *Mastomys* rodents, which are prevalent around human dwellings in West Africa, highlighting the interplay between human living conditions and disease spread.

Ebola and Marburg viruses, infamous for severe outbreaks in Central and West Africa, are primarily transmitted through contact with infected wildlife, particularly bats, or indirectly via contaminated bushmeat. Dense tropical forests facilitate these initial zoonotic transmissions, which are then spread among human communities through direct contact and worsened in healthcare settings that lack proper infection control measures. High human mobility, as especially evident during the 2014–2016 Ebola outbreak in Guinea, Liberia, and Sierra Leone, plays a key role in the rapid spread and persistence of the disease [54]. This outbreak, the largest since Ebola’s discovery in 1976, underscored major public health challenges in managing such diseases.

The outbreak also revealed instances of EVD recrudescence, where the virus, previously thought to be cleared, re-emerged in survivors. This was particularly concerning due to onward sexual transmission, which posed a significant risk for sparking new outbreaks. This aspect of the virus’s behaviour reveals the need for ongoing health services that extend beyond the immediate outbreak management. Research shows that Zaire ebolavirus can persist in immune-privileged sites such as the eyes and testes [55], and be transmitted through sexual contact months after a patient has recovered. This necessitates prolonged vigilance and support for survivors. These findings highlight the importance of comprehensive post-outbreak strategies that include continuous monitoring and community support to prevent new chains of transmission.

Another significant public health concern in West Africa, particularly affecting Sierra Leone, Liberia, Nigeria, and Guinea, is LF. These regions are home to *Mastomys natalensis* rodents, known carriers of the LASV that thrive in environments that are closely integrated with human dwellings [56]. This not only facilitates the spread of the virus from animals to humans, but also supports occasional human-to-human transmission [57]. Such transmissions are especially concerning in hospital settings lacking robust infection control measures as they more easily lead to outbreaks within the hospital, endangering patients, healthcare workers, and visitors. Such scenarios can amplify the spread of LF, making outbreaks more severe and challenging to contain.

Discovered in 1969, LF triggers regular seasonal outbreaks that intensify during the dry season when humans are more likely to come into contact with rodent habitats. Despite numerous recorded outbreaks, the largest and most severe occurred in 2018 [58], highlighting significant challenges in surveillance, diagnosis, and treatment across the affected regions. Significantly, this event underscored the critical need for ongoing research, enhanced preventive measures, and strengthened healthcare systems to effectively manage and mitigate the disease’s impact [59]. While the ongoing presence of LF in West Africa highlights the critical need for enhanced public health strategies and infrastructure to address this persistent threat, the virus also poses unique challenges due to its potential to be exported to non-endemic countries like the USA, UK, and other parts of Europe through international travel [60].

#### 2.2.2. The Most Notable VHFs in Europe Include CCHF and HFRS

CCHF has been reported in Albania, Greece, Kosovo, and Spain [61,62,63]. Recent evidence indicates that CCHFV is spreading in Europe, likely through migratory birds that carry infected ticks over long distances [64]. For instance, CCHFV has been detected in ticks found on migratory birds in Morocco and Spain, supporting the theory that the virus can be introduced into Europe by ticks transported from Africa. This spread is further facilitated by climate change, as longer and drier summers are causing Hyalomma ticks to move northward, increasing the potential range of the virus in Europe. The recent detection of CCHFV in southern France [65] potentially establishes new reservoirs and transmission cycles [66]. Migratory birds act as ecological bridges, enabling ticks, primarily *Hyalomma* spp., which are hardy and capable of surviving long journeys, to access geographically distant areas. This ecological dynamic poses significant challenges for disease surveillance and control, as many migratory routes can overlap with major human populations [67].

The predominant cause of HFRS in Europe is DOBV, transmitted through direct or indirect exposure to infected rodents (*Apodemus flavicollis*). The virus is found in several countries, but particularly Bosnia and Hercegovina, Croatia, Greece, and Slovenia. DOBV infections characteristically cause severe forms of HFRS compared to other orthohantaviruses, such as SEOV, which also circulates in Europe, and PUUV, responsible for a mild version of HFRS known as nephropathia epidemica.

#### 2.2.3. South America

In South America, various VHFs are closely linked to agricultural practices that enhance human contact with rodent vectors. Argentine haemorrhagic fever, caused by JUNV, is endemic to the agricultural pampas of Argentina, where it is transmitted through aerosolised particles or direct contact with infected rodents [68]. This region’s extensive farming activities facilitate the transmission of the virus, particularly during the maize-harvesting period when rodent populations peak and human exposure to these carriers is most likely. Similarly, Bolivian haemorrhagic fever, caused by MACV, and Brazilian haemorrhagic fever, caused by the SABV, are found in ecological conditions that support the proliferation of their respective rodent carriers. The CHAPV, identified in the Chapare region of Bolivia in 2003 [69], presents a new infectious threat notable for its potential for human-to-human transmission, as demonstrated during an outbreak affecting healthcare workers in 2019 [19]. The endemic nature of these diseases is exacerbated by environmental factors such as deforestation and agricultural expansion, which disrupt natural habitats and increase the proximity of rodents to human dwellings [70]. The association of these VHFs with specific regional and environmental conditions highlights the complex interplay between agricultural practices, ecological disruption, and human health risks in South America.

The *Choclo orthohantavirus* (CHOV), responsible for hantavirus pulmonary syndrome (HPS), is another significant threat in South America. It is closely associated with agricultural regions where humans come into contact with infected rodents, particularly in countries like Panama and Chile [71]. The virus is primarily transmitted through the inhalation of aerosolised rodent excreta, and human cases often coincide with periods of agricultural activity.

In addition to rodent-borne VHFs, vector-borne diseases like YF also pose a considerable threat in the region. Endemic to tropical areas of South America, YF is transmitted by *Aedes Haemagogus* and *Sabethes* mosquitoes, which thrive in both rural and urban environments [72]. Outbreaks of YF often occur when mosquito populations spike due to seasonal changes and human activities that create favourable breeding conditions, such as water storage in containers or deforestation.

#### 2.2.4. Asia

Asia presents a diverse landscape of VHFs linked to specific vectors and environmental conditions that influence their spread and impact. In addition to the well-documented Hantaviruses, such as HTNV, found predominantly in Eastern Asia, particularly in rural areas of China and Korea, which are notorious for causing severe renal and pulmonary syndromes, Asia also contends with other less common but equally concerning VHFs, such as AHFV, KFDV, and SFTSV. AHFV is primarily found in the Middle East, particularly Saudi Arabia, where it causes symptoms ranging from mild flu-like to severe haemorrhagic disease and neurological complications [73]. The proximity of humans to livestock and the prevalence of tick vectors contribute to its transmission. KFDV, endemic to South Asia, especially in forested areas of Karnataka, India, is transmitted by ticks infected by contact with numerous wild mammals. This virus was first identified in 1957 and continues to cause significant seasonal outbreaks almost every year. The disease is characterised by high fever, haemorrhagic symptoms, and a high mortality rate. Deforestation and encroachment into forested areas increase human exposure to tick habitats, thereby facilitating the spread of KFDV [43]. SFTSV is an emerging tick-borne virus first reported in China and subsequently in Japan and South Korea [74]. It causes severe fever, low platelet count, low white blood cell count, and gastrointestinal symptoms, often resulting is fatality rates as high as 30% [75]. The spread of this virus is facilitated by the expanding geographical distribution of susceptible tick vectors and reservoir hosts, with its increasing distribution associated with agricultural practices that enhance human–tick interactions [76,77]. In contrast, CCHFV has a wider geographic spread, extending across Asia from the Xinjiang region of China to the Middle East [78].

#### 2.2.5. North America

North America’s recorded cases of VHFs are mainly related to Hantaviruses, such as the SNV, which causes hantavirus pulmonary syndrome (HPS), a severe and often fatal cardiopulmonary haemorrhagic disease in humans. First identified during a 1993 outbreak of fatal respiratory illness in the Four Corners region of the USA, where the boundaries of New Mexico, Arizona, Utah, and Colorado meet, SNV has since been a subject of intense study and public health concern [79]. The virus is primarily carried by the deer mouse (*Peromyscus maniculatus*), a native rodent species widespread across North America and a habitat generalist known to opportunistically invade recently disturbed habitats [80]. Nevertheless, since the initial outbreak, New Mexico continues to lead the USA in the number of SNV cases, with 129 cases occurring between 1975 and 2023 and a mortality rate of 43% [81].

In addition to SNV, other hantaviruses have been identified in North America, including the *Bayou orthohantavirus* (BAYV) and *Black Creek Canal orthohantavirus* (BCCV), which are also capable of causing HCPS. BAYV is primarily found in the southeastern United States and is carried by the rice rat (*Oryzomys palustris*) [82], while BCCV, linked to the cotton rat (*Sigmodon hispidus*), has been detected in Florida [83]. Both viruses present similar clinical symptoms to SNV, including severe respiratory distress and high mortality rates, although cases are less frequent than those associated with SNV.

While hantaviruses are the primary VHFs in North America, other viral pathogens occasionally linked to haemorrhagic symptoms, such as certain strains of dengue virus, have also emerged in the southern parts of the continent, particularly in areas where Aedes mosquitoes are present. However, these cases are relatively rare compared to the impact of hantavirus infections.

The range of hantaviruses and their rodent hosts across North America underscores the importance of ongoing surveillance and public health interventions, particularly in regions with increased human–rodent contact, to mitigate the risk of future outbreaks.

### 2.3. The Role of Geographic Distribution and Ecology in VHF Diagnostics and Emergence

VHFs are caused by a diverse group of viruses, many of which are geographically restricted, often tied to specific ecological conditions involving animal reservoirs (such as rodents, bats, primates, or ungulates) and vectors like mosquitoes or ticks. Knowing the geographical distribution of these viruses is valuable for diagnostic work, especially for patients presenting with febrile illnesses following travel to endemic regions. For instance, VHFs such as Lassa fever and Ebola are prevalent in West and Central Africa, while South America experiences viruses like JUNV and MACV linked to agricultural activities. In North America, hantaviruses like SNV and BAYV are more common and allied to endemic rodent reservoirs.

Additionally, understanding the ecological relationships between wildlife reservoirs and vectors in these endemic areas not only aids in diagnosing and controlling current outbreaks, but also helps anticipate the emergence of VHFs in non-endemic regions. Factors like environmental changes, urbanisation, agricultural practices, and climate shifts could drive vectors and reservoirs into new territories, increasing the risk of these diseases becoming endemic in areas where they currently pose no threat. Therefore, surveillance in both endemic and non-endemic regions is important to identify and respond to these threats early.

## 3. Control and Prevention Strategies for VHFs

### 3.1. Overview of Vaccination Efforts

The control and prevention of VHFs present significant public health challenges due to the severity and rapid spread of these diseases. Vaccination is one of the most effective strategies for preventing and managing these outbreaks. Established vaccines for YF and Argentine haemorrhagic fever have proven effective, while vaccines for EVD have only recently made significant progress. Efforts to develop vaccines for other VHFs continue, although many still lack effective immunisation options. This discrepancy underscores the urgent need for continued research and development in vaccine technology to address these serious gaps.

**Yellow fever vaccine:** The YF vaccine is one of the oldest and most successful vaccines against a VHF. It was first developed in the 1930s, utilising an attenuation process that involved passing a wild-type Asibi strain through embryonic mouse and chicken tissue 176 times. This process produced the live-attenuated 17D vaccine strain [84], which is characterised by a loss of viscerotropism, neurotropism, and mosquito competence [85]. The vaccine is recognised as providing lifelong immunity, often exceeding 35 years, with a single dose, making it highly effective in the fight against YF [86]. The WHO recommends routine vaccination in all YF endemic countries and considers it an essential component of outbreak control in epidemic situations. Its widespread use in routine immunisation programs in Africa and Latin America has significantly reduced the incidence of the disease. However, despite its success, the 17D vaccine faces challenges in supply and demand, particularly during outbreaks, in which fractional dosing strategies have been employed to extend vaccine supplies. As a consequence of these issues in 2016, the WHO launched the “Eliminate Yellow Fever Epidemics” (EYE) initiative, aiming to reduce the burden of YF through enhanced vaccine coverage by distributing 1.3 billion doses to endemic regions by 2026 [87].

**Argentine haemorrhagic fever**, caused by JUNV, is effectively prevented by the Candid #1 vaccine. The vaccine was developed in the late 1970s, involving an adaptation of JUNV in guinea pigs and further passages in cultured cells [88]. It was licensed in Argentina in the 1990s after clinical trials demonstrated its high efficacy, exceeding 95%, and confirmed its safety profile, characterised by only mild side effects such as low-grade fevers and localised pain at the injection site [89]. Since its introduction, Candid #1 has greatly reduced the incidence of Argentine haemorrhagic fever, especially in high-risk areas, and has fundamentally changed the public health approach to managing this serious disease [90]. It is recommended for those living in or traveling to endemic areas and is part of a broader strategy that includes rodent control and public health education to avoid contact with potential rodent reservoirs. The ongoing success of Candid #1 emphasises the importance of targeted vaccine development and highlights the necessity of continuous surveillance and research to adapt to any changes in the virus’s behaviour or epidemiology. Such a proactive approach is essential for preventing outbreaks and ensuring the safety of populations at risk.

**Zaire ebolavirus vaccine:** The 2014–2016 West Africa EVD outbreak catalysed a massive acceleration in the refinement of EVD vaccines. Initial work using a recombinant vesicular stomatitis (rVSV) virus expressing the transmembrane glycoprotein of zaire ebolavirus was initially developed in 2005 [91]. During the 2014–1016 outbreak, this vaccine showed promising results in a phase III trial conducted in Guinea [92]. The vaccine was later used under a compassionate use protocol during the 2018 Eastern Democratic Republic of the Congo outbreak, and has since received regulatory approval in several countries [93] and licensure from the US Food and Drug Administration (FDA) and the European Medicines Agency (EMA) in 2019, under the brand name Ervebo. Following this, the manufacturers of the vaccine (Merck) gave permission to stockpile and potentially distribute to areas of need, particularly in Africa [94]. Additionally, a two-dose combination of Zabdeno (Ad26.ZEBOV), based on a recombinant adenovirus (Ad26) virus vector, and Mvabea (MVA-BN-Filo), based on a recombinant modified vaccinia Ankara virus vector platform, have been licensed and are also in use. The effectiveness and rapid deployment of these vaccines have been important in controlling recent outbreaks, showcasing a modern success story in VHF vaccination efforts.

**Hantavirus vaccines:** Over recent decades, various approaches have been explored for vaccine development, particularly targeting the most common hantavirus strains prevalent in SE Asia, such as HTNV. Hantavax is a commercialised inactivated vaccine, used for preventing HFRS in South Korea since 1990 [95]. While Hantavax has been available for decades, its use is limited to specific regions, and its effectiveness outside of those regions remains unclear. In the United States, a DNA-based hantavirus vaccine, designed to protect against multiple hantavirus strains, has shown useful promise [96], including the successful completion of Phase II clinical trials.

**Lassa fever vaccine:** Unlike YF EVD and HTNV, there is no widely used vaccine for Lassa fever, although several candidates are in development [97,98,99]. The genetic diversity of the LASV poses a significant challenge, necessitating a vaccine that remains effective across various genetic clades [100]. Recent advances in virology, genetic engineering, and immune profiling are contributing to the development of more potent vaccine candidates, aiming to offer broad protection against different LASV strains. These efforts are crucial in creating a vaccine that can adapt to the virus’s variability and provide effective prevention against this disease [101].

**Crimean–Congo haemorrhagic fever (CCHF) vaccine:** Efforts to develop a CCHF vaccine have included various approaches, such as inactivated viruses, DNA vaccines, and vector-based platforms. Research to identify potential antigenic targets within the virus’s structure has focused on the glycoprotein that elicits an immune response in the host [102], although the virus’s nucleoprotein has also been shown to be immunogenic and protective [103]. Among the most promising developments has been the use of a modified Ankara (MVA) vector to deliver key elements of the CCHF virus glycoprotein. This method has shown potential in preclinical trials, inducing specific immune responses that could provide protection against the virus [104]. Additional studies have explored the use of recombinant adenovirus vectors, subunit vaccines, plant-expressed platforms, virus-like particles, and mRNA-based candidates that incorporate CCHFV recombinant glycoproteins and/or nuclear proteins in order to stimulate a protective immune response [105]. Despite such developments, there is still no licensed vaccine available for CCHF. The ongoing research faces numerous hurdles, including limited funding and the complexity of conducting trials in regions where CCHF is endemic [78,106]. Continued efforts are necessary to move from laboratory research to clinical trials and to carefully assess the efficacy and safety of these candidates in human populations.

**Dengue vaccine:** Dengue is a significant global health concern, with an estimated 390 million infections annually across more than 100 countries. Efforts to develop a dengue vaccine began in the mid-20th century, but progress has been slow due to the complexity of the disease process and the co-circulation of the virus’s four serotypes. The challenge has been to create a vaccine that provides similar levels of immunity to all serotypes, as secondary infections with a different serotype often lead to severe forms of the disease. After decades of research, Sanofi Pasteur’s CYD-TDV (Dengvaxia) was approved in 2015, becoming the first licenced vaccine for dengue [107]. However, while Dengvaxia is effective in preventing severe dengue in individuals who have previously been infected. It increases the risk of severe disease in those who are seronegative, leading to strict guidelines recommending its use only in endemic areas and for those with prior dengue exposure [108]. While this vaccine marked a major breakthrough, the risks for seronegative individuals highlight the need for the further development of safer, more universally applicable vaccines.

### 3.2. Overview of Antiviral Treatments

While vaccines play a crucial role in the prevention of VHFs, antiviral treatments are essential for managing and mitigating the impact of these diseases, especially during outbreaks. The development and deployment of effective antiviral therapies can significantly reduce the morbidity and mortality associated with VHFs.

**Ribavirin**, a nucleoside analogue mimicking the structure of nucleosides, can be incorporated into viral RNA, whereby it induces mutations during replication, hindering the virus’s ability to proliferate and causing a decrease in viral load. Multiple reports have shown it to have efficacy against several VHFs, particularly Lassa fever [109]. However, a recent study that compiled a wide range of clinical data determined that the evidence for ribavirin against Lassa fever is in fact poor [110]. Similarly, while ribavirin has been reported to be effective against CCHFV in vitro [111], discordant results have been reported from multiple clinical studies [112,113], including clinical studies that determined it was ineffective [114]. 

**Pyrazinecarboxamide Compounds**, like favipiravir (T-705), T-1105, and T-1106, function by inhibiting the RNA-dependent RNA polymerase of RNA viruses, which blocks viral replication. These compounds have shown good activity in animal models for JUNV, RVFV, and YFV [115].

**Monoclonal Antibodies** target viral glycoproteins, neutralizing viruses by binding to their surface proteins and preventing entry into host cells. Monoclonal antibodies (mAbs) have emerged as a significant therapeutic strategy against VHFs, particularly Zaire ebolavirus. The most notable among these is the mAb cocktail known as ZMapp, which includes three different monoclonal antibodies that bind to the surface glycoproteins of the Zire ebolavirus. Clinical trials and subsequent FDA approvals of these treatments were accelerated in respect to the urgent need for effective therapies during the large EVD outbreak in West Africa from 2014 to 2016 and a subsequent EVD outbreak in the DRC in 2018, which provided a unique opportunity to conduct extensive clinical trials in real-world settings. As a result of these efforts, mAbs like ZMapp, and later REGN-EB3 and mAb114, underwent expedited review and were used effectively to treat patients during these outbreaks. Such experiences underscored the potential of mAbs in managing VHFs and paved the way for further research and development [116]. In 2020, the FDA approved Inmazeb [117], a combination of three monoclonal antibodies (atoltivimab, maftivimab, and odesivimab), making it the first FDA-approved treatment specifically for Zaire ebolavirus infection, showcasing a significant milestone in VHF therapeutics rooted in the lessons learned during recent outbreaks.

The development of mAbs as antiviral therapies for VHFs has shown remarkable promise, particularly in the fight against EVD, and similar approaches are being explored for other VHFs, such as LASV [118], CCHFV [119], and dengue [120].

The high-throughput screening of small molecular libraries has emerged as a promising way to identify new candidate antivirals for VHF therapy [121]. A promising small molecule that has been identified in this way, initially against Zaire ebolavirus, is FGI-106 [122]. Interestingly, its antiviral activity extends across a diverse range of VHF viruses, including RVFV, HTNV, ANDV, and CCHFV [123].

### 3.3. Importance of Surveillance and Early Detection

Surveillance and early detection play pivotal roles in controlling and mitigating the impacts of VHFs, being essential for the timely identification of outbreaks and enabling rapid public health responses designed to reduce the morbidity and mortality associated with these diseases. Robust surveillance systems help track the spread of VHFs, monitor their evolution, and provide critical data that inform public health interventions and strategies [124]. The importance of surveillance in the management of VHFs is underscored by the often rapid and unpredictable nature of these outbreaks. Effective surveillance systems can provide early warning signs that are crucial for preventing widespread transmission [125]. These systems integrate data from various sources, including healthcare facilities, laboratories, and field reports, to achieve a comprehensive understanding of outbreak dynamics [126]. The early detection of VHFs is equally important. It relies on the capacity of health systems to diagnose cases swiftly and accurately. Advances in diagnostic technologies have greatly enhanced the ability to detect VHFs early in the course of an outbreak. Rapid diagnostic tests (RDTs) and point-of-care technologies now enable health workers in remote areas to diagnose diseases quickly, facilitating immediate containment and treatment measures [127]. The integration of digital tools and mobile technology into surveillance networks has also transformed the landscape of disease monitoring. These technologies allow for real-time data collection and analysis, which is vital for tracking the movement of viruses and their vectors across different geographies [128]. Furthermore, geographic information systems (GIS) and remote sensing technologies provide detailed insights into the environmental factors that influence the spread of VHFs [129]. Together with advanced portable technologies, such as the MinION device for real-time genomic sequencing, surveillance and control approaches for VHFs can be enhanced [130], aiding the prediction and prevention of outbreaks.

Global cooperation and data sharing are essential components of effective surveillance and early detection systems. Countries less frequently affected by viral haemorrhagic fevers often encounter challenges in rapidly identifying and diagnosing these diseases due to limited exposure and unfamiliarity among healthcare providers. This can result in a delayed implementation of critical isolation protocols and infection control measures, heightening the risk of local transmission. Furthermore, these health systems face the ongoing challenge of maintaining readiness for such severe pathogens, necessitating continual training and the equipping of health personnel to safely and effectively manage potential cases. The importance of global health security measures, including robust disease surveillance systems and international collaboration on outbreak response, to prevent the spread of VHFs beyond their usual regions is clear [131]. International health regulations and networks such as the World Health Organisation’s Global Outbreak Alert and Response Network (GOARN) facilitate the exchange of information and resources among countries, enhancing global capacity to manage VHFs. This collaborative approach is central in the interconnected world where the movement of people and goods can quickly turn a local outbreak into a global health emergency [132].

## 4. Challenges in Studying VHF Viruses and Animal Vectors

Studying VHF viruses and their animal vectors presents a complex set of challenges that stem from the viruses’ diverse nature, their varied ecosystems within which they thrive, and the multifaceted interactions between hosts, vectors, and environmental factors. These challenges significantly impact research, surveillance, and our overall understanding of VHFs, necessitating an interdisciplinary approach to elucidate and ultimately overcome these issues.

### 4.1. Obstacles in Research and Surveillance

The high degree of genetic diversity in VHF viruses significantly complicates the development of effective vaccines and diagnostic tools. This diversity influences how these viruses interact with their hosts and vectors, affecting the dynamics of disease transmission and the patterns of outbreaks. Moreover, their continual evolution presents ongoing challenges as researchers strive to keep pace with new strains and mutations. Basic research into VHFs is also constrained by the need for high-containment laboratories, which are required due to the viruses’ high pathogenicity and the severe risks they pose to human health. Constructing and maintaining such facilities is not only costly, but these laboratories are also limited in number globally, a constraint which significantly restricts the opportunities for direct, hands-on research with these dangerous pathogens.

Field studies on VHFs pose their own set of challenges, particularly in remote and often politically unstable regions where these viruses are typically found. A lack of infrastructure and limited healthcare capabilities add to these general difficulties, complicating data collection and safety assurances for researchers. Despite these obstacles, field studies are essential for gaining a deep understanding of VHF dynamics within their natural environments. Handling live viruses and infected vectors during research also raises considerable ethical and safety concerns. Moreover, transporting samples across international borders for research purposes involves navigating complex regulatory landscapes, potentially delaying crucial research and response efforts.

### 4.2. Interdisciplinary Approaches in VHF Management

Understanding and managing VHFs necessitates a robust, interdisciplinary approach that combines insights from virology, ecology, zoology, and epidemiology. This integration of specialties is key to assessing how environmental changes, such as deforestation and climate change, impact vector behaviour and interactions between humans and animal reservoirs. Such a perspective enables more accurate predictions and tailored interventions to mitigate the spread of these diseases.

Effective VHF surveillance and control also require international cooperation, bridging gaps between various scientific disciplines and public health initiatives. By pooling knowledge, resources, and strategies on a global scale, the international health community can forge comprehensive and pre-emptive responses to VHF outbreaks. Employing advanced technologies like GIS, remote sensing, and genomic sequencing enhances the capability to monitor and analyse the spread and transmission patterns of VHFs in real time. Such tools are key to gathering data and generating insights that drive effective public health interventions.

Moreover, local community engagement plays a critical role in the successful implementation of surveillance and control measures. Here, understanding local cultural practices and perceptions of disease is key to designing interventions that are not only effective, but also culturally sensitive and accepted. Educational campaigns that focus on preventive measures and increase symptom awareness are essential to reduce the risk of VHF transmission, highlighting the need for an integrated approach that combines scientific research with community-based strategies. This comprehensive framework not only addresses the biological and ecological aspects of VHFs, but also incorporates the social dimensions essential for managing public health threats effectively.

## 5. Conclusions and Future Directions

Recurring outbreaks of VHFs urgently call for improved surveillance, rapid diagnostic capabilities, and effective public health response strategies. The challenges in surveillance, diagnosis, and treatment highlight the importance of continued research, improved preventive measures, and strengthened healthcare responses. These complexities demand a concerted effort from the scientific and global health communities to expand the frontiers of interdisciplinary research and collaboration. Looking ahead, several key areas will be pivotal in shaping the future management and research of VHFs:

**Enhanced Global Collaboration**: As VHFs continue to pose threats across borders, enhanced international cooperation is critical. This includes not only the sharing of surveillance data, research findings, and healthcare strategies. but also investment in capacity building. Collaborative networks like the WHO’s Global Outbreak Alert and Response Network (GOARN) play a key role, but further expansion is needed to ensure global responsiveness to VHF outbreaks. Developing equitable collaborations requires investment in training programs, such as PhD scholarships for scientists and healthcare workers in low- and middle-income countries (LMICs) where VHFs and arboviruses are endemic. This fosters local expertise and ensures balanced contributions to global research and outbreak response efforts. Access to resources, technology, and opportunities, along with the transparent sharing of intellectual property and fair funding distribution, are key to creating equitable partnerships. These efforts not only enhance global VHF responses, but also ensure that knowledge and capacity remain sustainable in vulnerable regions.

**Advances in Technology**: The application of new technologies such as genomic sequencing and remote sensing should continue to be expanded. These tools offer real-time insights into the spread of viruses and can significantly enhance our understanding of outbreak dynamics. Investing in portable diagnostic and field validation technologies that can be used in remote or resource-limited settings will be particularly useful.

**Interdisciplinary Research**: The ecological and biological complexities of VHFs demand a continued interdisciplinary approach that merges a growing range of scientific disciplines. Developing a better understanding of the interplay between viral host reservoirs, hematophagous arthropod vectors, environmental changes, and basic virus biology is key for devising effective prevention and control strategies.

**Community Engagement**: The effective management of VHFs also depends on local community involvement. Tailoring public health messages and interventions to fit local cultural contexts can greatly enhance their effectiveness. Moreover, increasing local capacities in surveillance and response not only empowers communities, but also leads to more sustainable health outcomes.

**A One Health Focus**: Given the zoonotic nature of many VHFs, the One Health approach—which recognises the interconnectedness of the health of people, animals, and ecosystems—remains a vital framework and can help guide research, policymaking, and education to foster an inclusive understanding and management of VHFs.

**Policy and Infrastructure Development**: Strengthening health infrastructure, especially in endemic regions, and developing policies that support robust surveillance and rapid response capabilities are essential to improving diagnostic capabilities, healthcare access, and emergency response systems.

**Vaccine Development and Deployment**: There is an urgent need to advance the research and development of vaccines for VHFs, particularly in areas where no effective vaccines currently exist. Prioritising innovations in vaccine technology and delivery systems that tackle the unique challenges posed by VHFs is essential. The licensing of VHF vaccines faces numerous challenges, including the sporadic and geographically limited nature of outbreaks, which complicates the conduct of comprehensive clinical trials. Additionally, the substantial resources required to bring a vaccine to licensure should not be underestimated. Once these hurdles are overcome, it is then imperative to ensure the equitable distribution of vaccines, particularly in resource-limited settings.

**Sustainable Funding Models**: Ensuring continuous and predictable funding for VHF research and outbreak response is important because strengthening surveillance systems, and thus enabling rapid responses to outbreaks, ultimately helps to mitigate the public health impact of VHFs and prevent future epidemics. This includes supporting basic scientific research, as well as funding for the implementation of control measures and healthcare interventions in outbreak-prone areas. These models should prioritise long-term investments in healthcare infrastructure, disease surveillance, and prevention programs, rather than relying solely on emergency funds triggered by outbreaks. To ensure equitable global collaboration, it is important that funding is distributed fairly, particularly to LMICs that bear the brunt of VHF outbreaks. This includes financing for local training programs, capacity building, and healthcare interventions in affected regions, allowing these countries to play an active role in global research and response efforts. Furthermore, public–private partnerships may help diversify funding sources, combining resources from governments, international organisations, philanthropic institutions, and the private sector. Bringing together diverse expertise, resources, and perspectives from multiple sectors via these partnerships fosters innovation and can help maintain steady funding streams to cover gaps in traditional models.

By addressing these areas, the global health community can enhance its readiness and capacity to manage and mitigate the impacts of VHFs. The path forward requires a sustained commitment to research and development, proactive surveillance, international cooperation, and community-centred interventions. Only through a concerted global effort can we hope to control and eventually prevent the spread of these severe and often devastating diseases.
